# Cellular landmarks of *Trypanosoma brucei* and *Leishmania mexicana*

**DOI:** 10.1016/j.molbiopara.2018.12.003

**Published:** 2019-06

**Authors:** Clare Halliday, Karen Billington, Ziyin Wang, Ross Madden, Samuel Dean, Jack Daniel Sunter, Richard John Wheeler

**Affiliations:** a Sir William Dunn School of Pathology, University of Oxford, South Parks Road, Oxford, OX1 3RE, UK; b Department of Biological and Medical Sciences, Oxford Brookes University, Gipsy Lane, Oxford, OX3 0BP, UK; c The Peter Medawar Building for Pathogen Research, University of Oxford, South Parks Road, Oxford, OX1 3SY, UK

## Abstract

The kinetoplastids *Trypanosoma brucei* and *Leishmania mexicana* are eukaryotes with a highly structured cellular organisation that is reproduced with great fidelity in each generation. The pattern of signal from a fluorescently tagged protein can define the specific structure/organelle that this protein localises to, and can be extremely informative in phenotype analysis in experimental perturbations, life cycle tracking, post-genomic assays and functional analysis of organelles. Using the vast coverage of protein subcellular localisations provided by the TrypTag project, an ongoing project to determine the localisation of every protein encoded in the *T. brucei* genome, we have generated an inventory of reliable reference organelle markers for both parasites that combines epifluorescence images with a detailed description of the key features of each localisation. We believe this will be a useful comparative resource that will enable researchers to quickly and accurately pinpoint the localisation of their proteins of interest and will provide cellular markers for many types of cell biology studies. We see this as another important step in the post-genomic era analyses of these parasites, in which ever expanding datasets generate numerous candidates to analyse. Adoption of these reference proteins by the community is likely to enhance research studies and enable better comparison of data.

## Highlights

• *Trypanosoma* and *Leishmania* are single cell eukaryotic parasites.
• The cell organisation of these human pathogens is complex and highly structured.
• This describes an inventory of reliable reference markers for 32 cell structures.
• These light microscopy landmarks are a valuable resource for researchers.

## Graphical abstract

**Figure:**
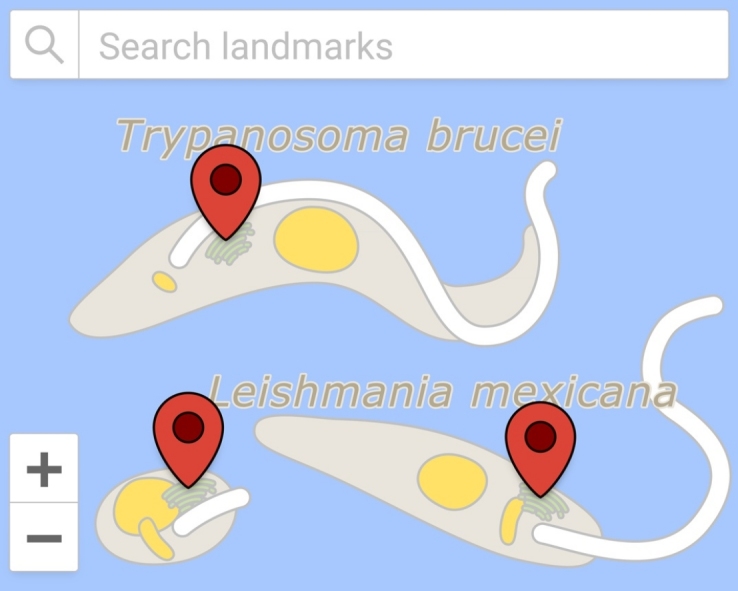

## 1 Introduction

**Fig. 1:**
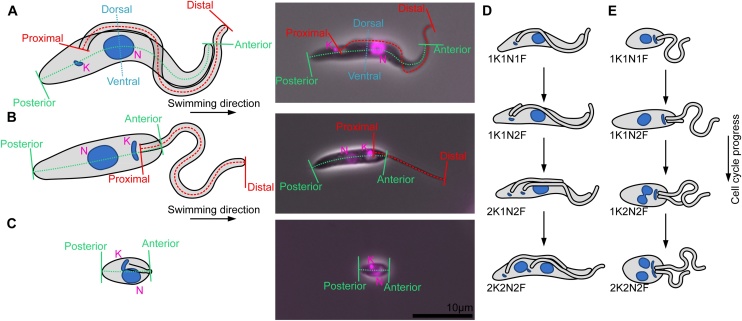
The morphology of *T. brucei* and *L. mexicana*. A–C. The morphologies of key culturable life cycle stages of *T. brucei* and *L. mexicana*, shown in cartoon form (Left) and as an overlay of a phase contrast and Hoechst (DNA stain) fluorescence micrographs (Right). **A**. Procyclic form *T. brucei* with a trypomastigote morphology. The anterior-posterior axis, the kinetoplast (K) and nucleus (N), the flagellum proximal-distal axis and the dorsal-ventral axis are indicated. **B**. Procyclic form *L. mexicana* with a promastigote morphology. No features visible by light microscopy can be used to define a dorsal-ventral axis. **C**. Amastigote form *L. mexicana*. The flagellum does not protrude from the cell, meaning a flagellum proximal-distal axis is not easy to identify. **D**–**E**. The key cell cycle stages of procyclic form *T. brucei* and *L. mexicana,* showing the order of duplication of the kinetoplast (K), nucleus (N) and flagellum (F) and their morphology. **D**. Procyclic trypomastigote form *T. brucei*. **E**. Procyclic promastigote form *L. mexicana.*

Over the last 30 years, a set of tools and technologies has been developed to enable the imaging of protein localisations in the kinetoplastid parasites, including monoclonal antibodies, epitope tags and most recently fluorescent protein tags [[1], [2], [3], [4], [5], [6]]. Given that the kinetoplastids such as *Trypanosoma brucei, Trypanosoma cruzi* and *Leishmania* spp. are highly structured polarised eukaryotic cells, a microscope image of the subcellular pattern of signal from immunofluorescence or fluorescent protein tagging is critical step for elucidating phenotype analysis or the potential protein function. It is often possible to determine which organelle or organelle sub-structure a protein localises to by reference to key landmarks observed by phase contrast microscopy and detection of nuclear and kinetoplast landmark positions via fluorescent DNA staining (Fig. 1). A standard set of references to which comparisons could be made would therefore be useful to the field. Here, we report a collection of reference protein localisations for *T. brucei* and *Leishmania mexicana* (as a representative *Leishmania* species). We used the commonly cultured forms of these parasites, the insect gut forms (procyclic and promastigote) as well as the amastigote (mammalian macrophage-inhabiting) form of *L. mexicana*.

A procyclic *T. brucei* cell has a trypomastigote [7] morphology: An elongated cell body that tapers at both ends with a long flagellum that is laterally attached to the side of the cell body for the majority of the length of the flagellum. The tips of the long cell body, the flagellum and the flagellar pocket constitute the key landmarks of the cell by phase contrast or differential interference contrast (DIC) light microscopy. These structures create clear asymmetries that allow the definition of a series of reference axes (Fig. 1). The anterior-posterior axis is defined by the direction of swimming, with the flagellum extending beyond the anterior end of the cell body. The dorsal-ventral axis is defined by the lateral attachment of the flagellum to the cell body with the dorsal side marked by the flagellum attachment zone [8]. Finally, the proximal (base) to distal (tip) axis along the flagellum provides the third reference axis.

A promastigote *Leishmania* cell has an elongated cell body that is rounded at the cell end from which the long flagellum emerges and tapered at the opposite cell end [7]. As with trypanosomes the anterior-posterior axis is defined by the direction of swimming, with the flagellum emerging from the anterior end of the cell (Fig. 1). By phase contrast, the *Leishmania* cell body appears rotationally symmetric around its anterior-posterior axis so it is difficult to define a dorsal-ventral axis. However, the proximal-distal axis along the flagellum can provide a further reference axis for *Leishmania*.

*Leishmania mexicana* promastigotes can be differentiated *in vitro* to form axenic amastigotes that are similar to intracellular amastigotes found inside the parasitophorous vacuole of infected macrophages during a mammalian infection [9]. These cells have an amastigote morphology: An ovoid cell body from which a short flagellum just emerges at the anterior end. *Leishmania* amastigotes are immotile and therefore the anterior-posterior axis is defined by analogy to the promastigote form and follows the proximal-distal axis along the flagellum. As with the promastigote form, the amastigote cell body appears rotationally symmetric around its anterior-posterior axis so it is difficult to define a dorsal-ventral axis using phase-contrast microscopy.

In addition to the cell shape landmarks that are easily visible by phase contrast or DIC, the positioning of the DNA containing structures, the nucleus and kinetoplast (concatenated mitochondrial DNA), are consistent and predictable (Fig. 1). Therefore, the combination of a phase contrast image and fluorescent DNA stain image provides an ideal reference framework for determining protein localisation. Moreover, the timing of the duplication and division of the nucleus, kinetoplast and cytoskeletal features (most strikingly, the flagellum) occur at set time points during the cell cycle. A simple count of these features enables the cell cycle stage of any cell to be determined and hence allows proteins with cell cycle dependent expression or localisation patterns to be identified (Fig. 1) [[10], [11], [12]].

TrypTag is an ongoing project which is successfully generating a subcellular localisation database of every protein encoded in the *T. brucei* genome [13]. This project is building on these inherent cell biological advantages to build a localisation database of high biological value for many fields. Once complete, the data set will be in two parts: firstly, the images of the trypanosome cell expressing the tagged protein and, secondly, the annotation assigning that protein to a likely subcellular localisation. The annotation of the images is of particular importance as this enables researchers to search for proteins with a specific localisation. Consideration of the many protein localisations obtained so far shows that some provide extremely clear, reproducible markers for organelles. We have therefore developed this resource using well-characterised proteins as a reference for the majority of organelles and organelle sub-domains in the cell to guide our annotation of the localisations observed during the TrypTag project. This resource shows that it is possible, with care, to distinguish between localisations that can appear superficially similar. To add comparative value, we have also localised these proteins in *L. mexicana* as a representative *Leishmania* species. However, this resource will also provide an important reference for other parasite cell biology communities. Widespread use of cell lines expressing these standard markers described here will facilitate meta-analyses over the coming years and provides a foundation for analysis of changes in structure in both trypanosomatid mutants and different life cycle stages.

## 2 Results and discussion

This resource provides illustrative widefield epifluorescence images of proteins endogenously tagged with a fluorescent protein which localise to specific structures/compartments in the *T. brucei* and *Leishmania* cell. This is supported by a description of key features distinguishing these localisations, a localisation ontology (a defined vocabulary) to describe them and the associated Gene Ontology (GO) cellular component accession numbers of the structure. In collaboration with TriTrypDB we have submitted GO definitions to allow the pairing of all localisation ontology terms with GO terms, although not all structures have yet been assigned GO terms.

Wherever possible, example proteins have been selected which are major components of a structure/compartment and for which there is previously published evidence for localisation to that structure/compartment. In some cases, where that was not possible, we have used proteins either with well-characterised orthologs in other organisms or with well-known biochemistry. For these cases we have indicated if this protein has a subcellular localisation known in either the yeast or human genome-wide subcellular protein localisation projects [14,15]. If no example protein is given then it means that to date there is no previously described example in trypanosomatids nor any ortholog from another organism with the expected localisation.

The localisation descriptions are designed to be used in an additive manner; therefore, if the fluorescent pattern from a tagged protein shows that protein is localised to more than one organelle then all the appropriate descriptors should be listed. Moreover, for the more complex organelles that contain sub-domains we have arranged the descriptions within a hierarchy reflecting their position within that organelle; for example, the nuclear pore is a component of the nuclear envelope, which in turn is part of the nucleus. We have indicated this hierarchy using numbered headings.

This ontology presented here provides a defined set of terms useful to the kinetoplastid community for future descriptions of cellular localisations. There follows in most cases a nominated marker protein defining that organelle/structure. Sometimes there is a GO term that is useful for describing the general location of a novel protein where a specific sub-definition in the hierarchy has not been determined – e.g. nucleus. Whilst we include these more generic terms for completeness we have not ascribed markers to them, since they are generally too granular to be useful. In looking at the many thousands of tagged proteins in the TrypTag project we have chosen a subset cohort whose localisation provides an inventory of organelles and structures useful for studies in trypanosomes and *Leishmania*.

**Fig. 2:**
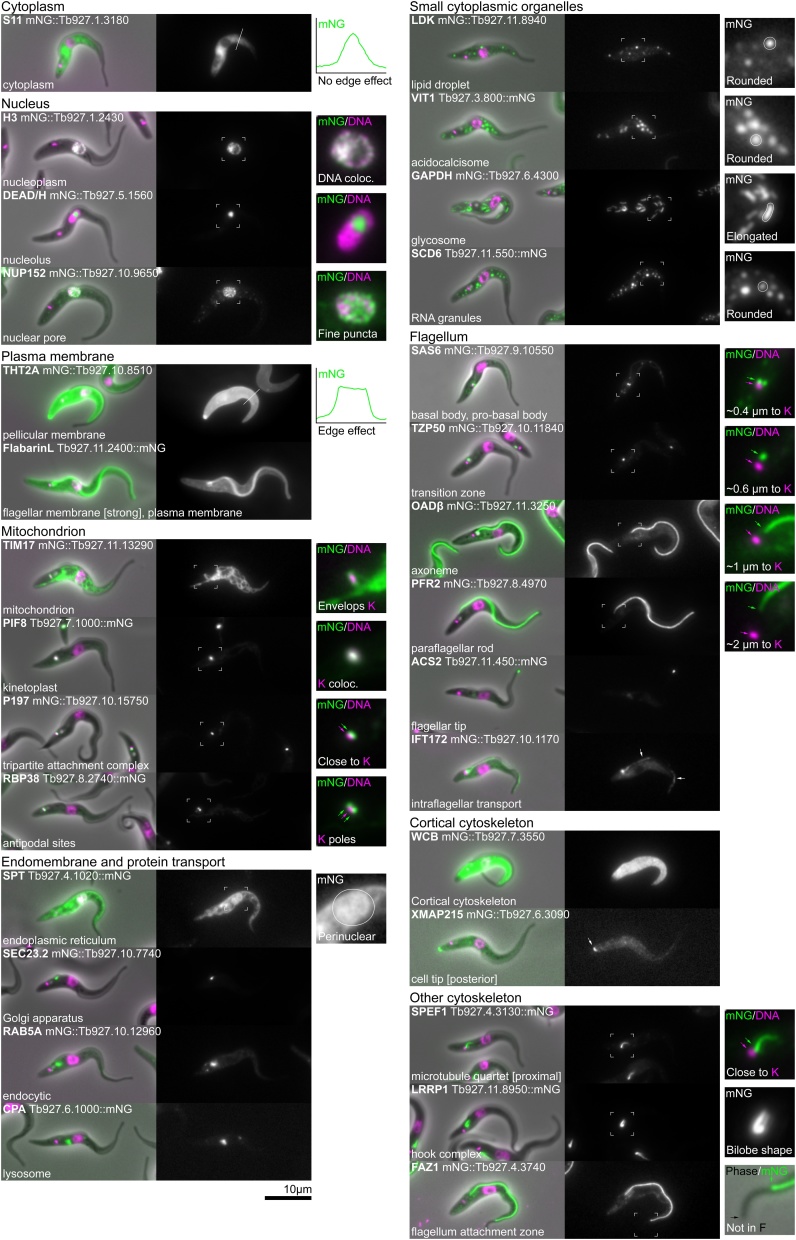
Reference protein localisations for procyclic trypomastigote form *T. brucei*. Widefield fluorescence images for each protein are laid out in the same format: Left, an overlay of the phase contrast (grey), mNG fluorescence (green) and Hoechst DNA stain (magenta) and right, the mNG fluorescence in greyscale. These images were all captured as part of the TrypTag project. The protein name and gene fusion are shown in the top left (Tb927.X.XXXX::mNG for C terminal tagging, mNG::Tb927.X.XXXX for N terminal tagging). The annotation of the localisation is shown in the bottom left. A key distinguishing feature of the localisation may be highlighted on the right.

**Fig. 3:**
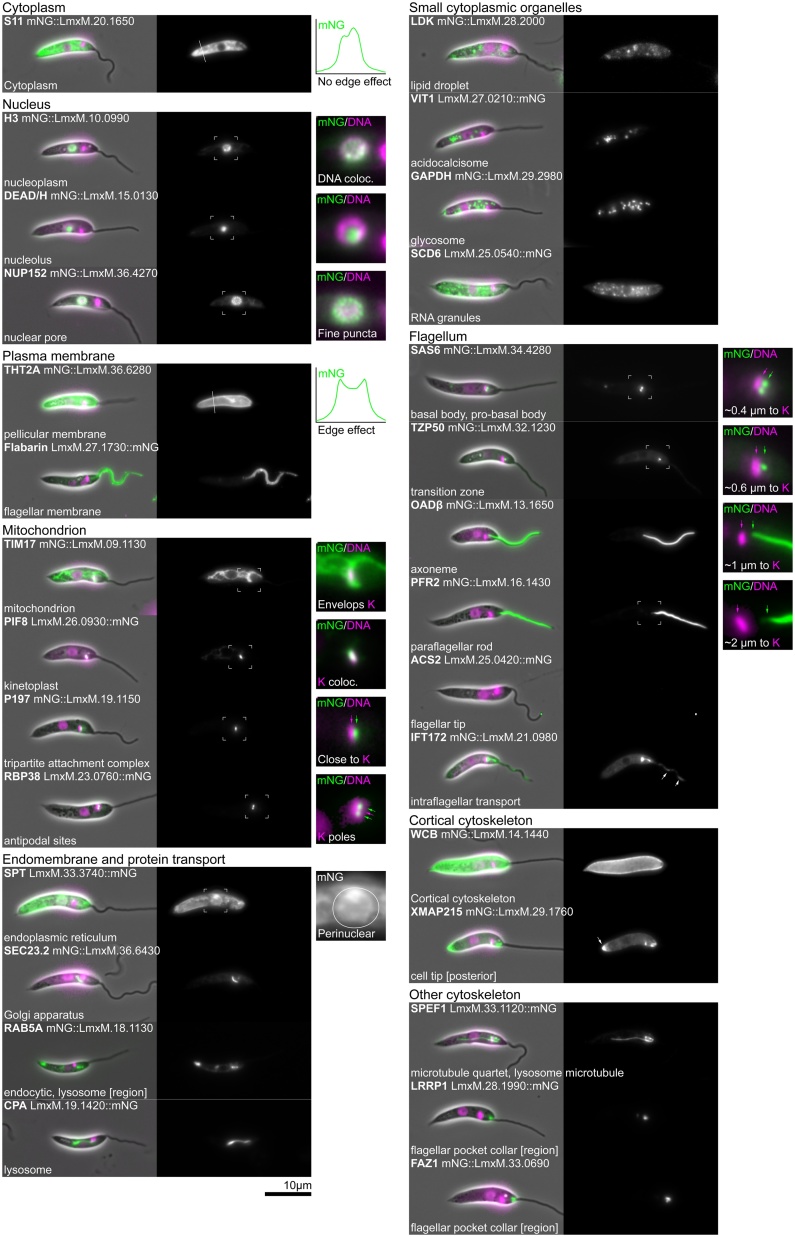
Reference protein localisations for promastigote procyclic *L. mexicana*. Widefield fluorescence images of the localisation of the *L. mexicana* orthologs of the proteins shown in Fig. 2. Localisations are presented in the same order and using the same layout as for *T. brucei* for easy comparison.

**Fig. 4:**
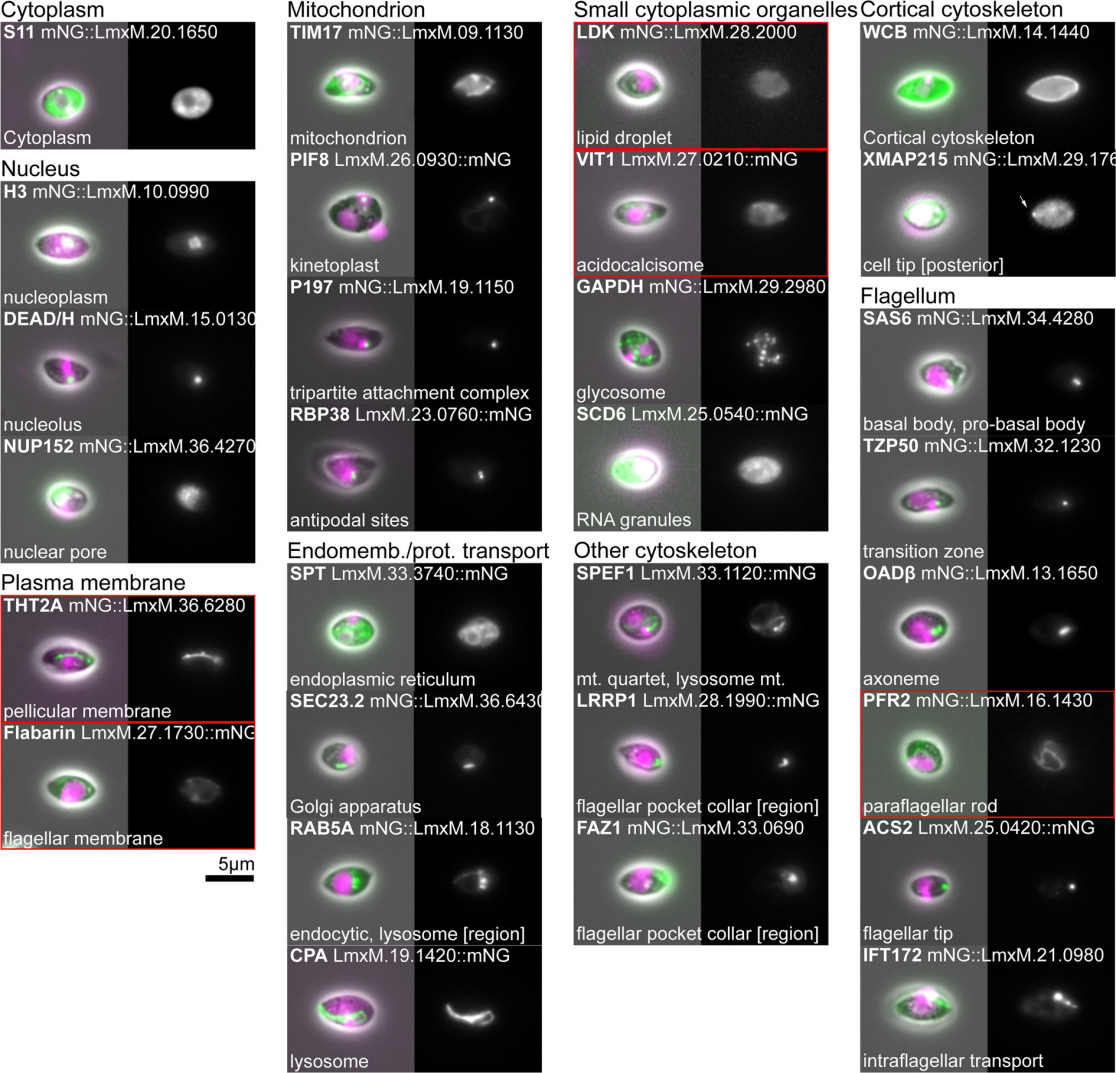
Reference protein localisations for axenic amastigotes *L. mexicana*. Widefield fluorescence images of the localisation of the *L. mexicana* proteins shown in Fig. 3. Localisations are presented in essentially the same order and using the same layout as Fig. 2, Fig. 3. Image contrast for fusion proteins not expressed in the amastigote approximately matches the contrast in Fig. 2. Red outlines indicate a localisation that may be spurious, see main text for more detail.

**Fig. 5:**
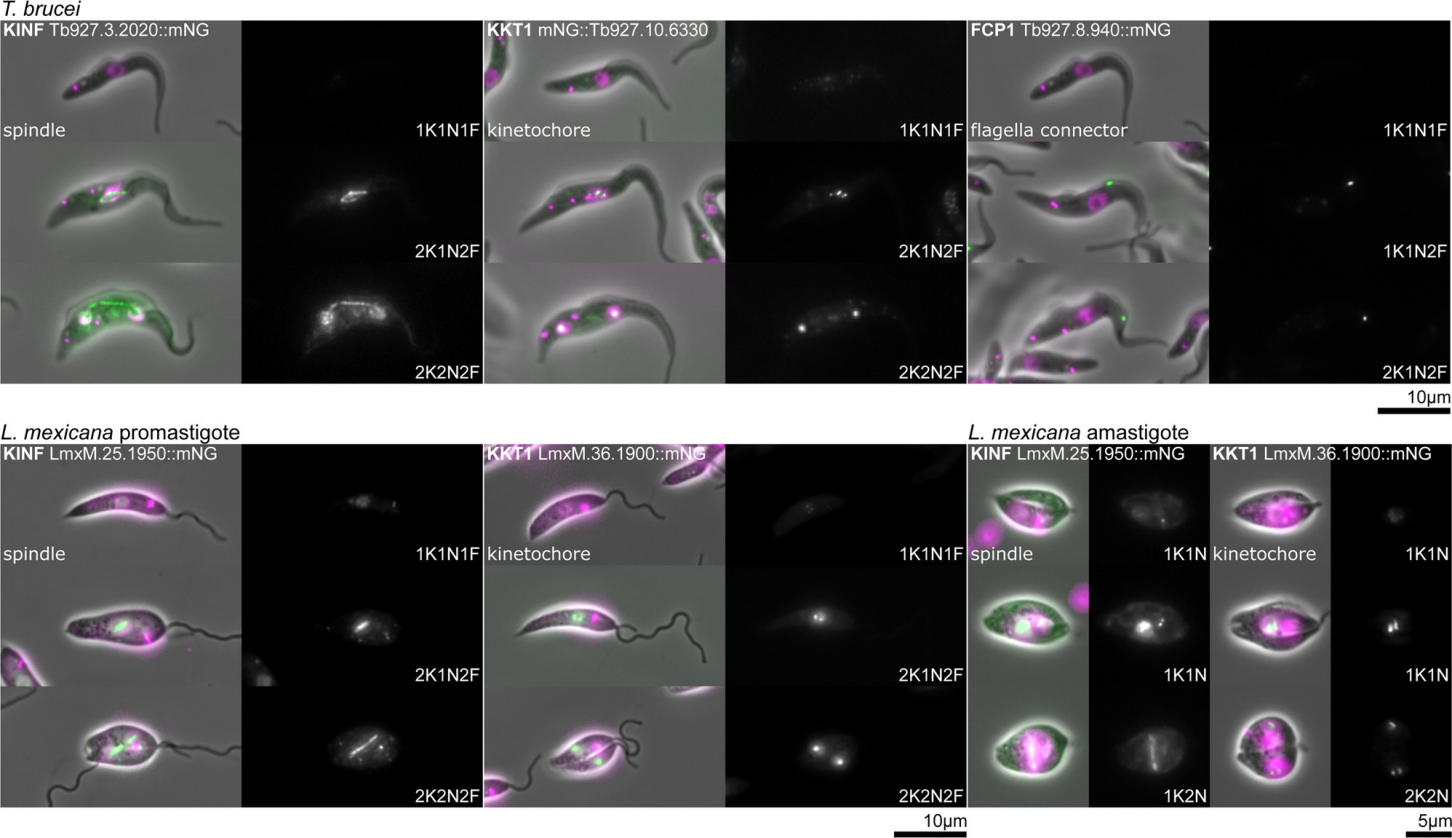
Cell cycle dependent localisations in *T. brucei* and *L. mexicana*. Widefield fluorescence images of key cell cycle dependent localisations in *T. brucei* procyclic trypomastigotes and *L. mexicana* procyclic promastigotes and amastigotes. For each protein localisation the number of kinetoplasts (K), nuclei (N) and (where visible) flagella (F) are indicated. Image contrast is the same for each cell cycle stage. *L. mexicana* amastigotes had few dividing cells after 72 h differentiation, so the first amastigote morphology division (10 h after division) is shown.

Overall, the structure and organisation of many organelles and organelle sub-domains are similar between *T. brucei* and *L. mexicana*; however, there are certain structures such as the lysosome and flagellum attachment zone that differ significantly, and we highlight these differences. We have shown reference marker protein localisations in *T. brucei* procyclic trypomastigotes in Fig. 2, *L. mexicana* promastigotes and amastigotes in Fig. 3, Fig. 4 respectively and cell cycle dependent localisations in Fig. 5. Some amastigote cell lines gave a weak or ambiguous signal, which may have one of several causes (see below). We have drawn attention to this limitation in Fig. 4 by means of a red outline to the relevant micrographs.

Our tagging approach introduces the mNeonGreen [16] open reading frame into the endogenous locus of the target gene to allow expression of a protein with an amino (N) or carboxyl (C) fluorescent tag. For the reference images here, we have selected the terminus which gave the clearer localisation, assessed qualitatively based on signal and background intensity. For the majority of these proteins, tagging at either the N or C terminus gave the same localisation; however some protein localisations (a subset of plasma membrane, mitochondrion and endomembrane proteins) were sensitive to the tagging terminus. This endogenous tagging method uses ‘readthrough’ transcription such that the tagged proteins’ expression is more likely to reflect that of the wild type protein than when other commonly-used methods are employed, such as exogenous promoter-driven expression. Clearly, however, we and other users are aware that such endogenous tagging introduces an exogenous UTR either on the 5′ or 3′ end, depending on which terminus of the protein is tagged. This could lead to over- or mis-expression of a subset of proteins, especially for C terminal tagging because the 3′ UTR is thought to encode most of the regulatory signals for controlling gene expression [17]. Therefore, as with any such global approaches there are unavoidable caveats that will no doubt be born in mind by the user; however, in these examples we have been careful to locate the tag in each specific protein marker at the most appropriate terminus.

## 3 Nucleus - GO:0005634

*T. brucei* and *Leishmania* have a single, near-spherical nucleus located approximately in the centre of the cell, which undergoes closed mitosis. In *T. brucei* mitosis follows kinetoplast division [10,18,19], while in *Leishmania* mitosis and kinetoplast division occur near-synchronously [11,12]. The nucleus is readily identified using DNA stains and sub compartments are readily identifiable.

### 3.1 Nuclear lumen - GO:0031981

This is the entire membrane bound contents of the nucleus. A nuclear lumen protein localisation is identified from a nuclear signal without exclusion from the nucleolus and without an ‘edge effect’ (concentration of the signal at the edge of the nucleus) which would indicate a nuclear envelope signal. Many, if not most, nuclear proteins are concentrated in either the nucleoplasm or nucleolus.

### 3.2 Nucleoplasm - GO:0005654

This is the lumenal contents of the nucleus excluding the nucleolus, comprised of euchromatin and heterochromatin. A nucleoplasm protein localisation gives a nuclear lumen signal that is excluded from the nucleolus. **Example protein: Histone 3, H3** [20] (Fig. 3). Specialised nuclear bodies including the expression site body (ESB, the site of VSG mRNA transcription) are recognisable as single or multiple points in the nucleoplasm but are not readily distinguishable by light microscopy without co-localisation evidence; therefore, we have not described them here.

#### 3.2.1 Nucleolus - GO:0005730

Each nucleus has a single, near-spherical nucleolus responsible for ribosomal RNA synthesis which is visible as the region of lower signal intensity in the nucleus when viewed using a DNA stain. During mitosis the nucleolus does not break down [21] and instead becomes stretched along the spindle before resolving into two separate nucleoli as mitosis reaches completion. **Example protein: DEAD/H RNA helicase**, the ortholog of which localises to the nucleolus in humans [14] (Fig. 3).

#### 3.2.2 Spindle - GO:0005819

Spindle protein localisations are recognisable from the characteristic spindle structure: Diamond-shaped in early mitosis (within an elongated nucleus) or long and thin in late mitosis (between two nascent nuclei connected by a long bridge) [22]. The spindle is parallel to the anterior-posterior axis of the cell in *T. brucei* [22]. In *L. mexicana* the spindle begins near parallel to the anterior-posterior axis, before rotating to be near perpendicular [12]. As in many organisms which undergo closed mitosis, condensed chromosomes are not visible. **Example protein: Spindle-associated orphan kinesin F, KINF** [23,24] (Fig. 5).

##### 3.2.2.1 Spindle poles - GO:0000922

The ends of the spindles are bundles of microtubule minus ends and associated structures, although while microtubule bundling occurs in trypanosomes little is known about the associated structures [25]. Proteins localised to this structure give two signal foci, one on each of the outermost edges of nascent and recently divided nuclei – although they may hypothetically give more varied structures during spindle assembly or disassembly. These foci are separated along the anterior-posterior axis in *T. brucei* and separated perpendicular to the anterior-posterior axis in *Leishmania*. This structure is only present in mitotic cells with a spindle. No proteins unique to the spindle poles have been well characterised to date in the kinetoplastids.

##### 3.2.2.2 Kinetochore - GO:0000776

The kinetochores attach the centromere of chromosomes to the spindle microtubules and have recently been characterised in detail [26]. Proteins in this structure give a punctate signal in late pre-mitotic and mitotic nuclei, and often have decreased signal levels at other cell cycle stages. In early mitotic cells the points lie as a line perpendicular to the orientation of the spindle (analogous to the metaphase plate), before moving towards the spindle poles [26]. **Example protein: Kinetoplastid kinetochore protein 1, KKT1** [26,27] (Fig. 5).

### 3.3 Nuclear envelope - GO:0005635

This is the double membrane enclosing the perinuclear space that surrounds the nucleus and is contiguous with the endoplasmic reticulum. Proteins found in this structure would be expected to give a nuclear signal with a clear increase in signal around the periphery of the nucleus – an ‘edge effect’. However, to date, no nuclear envelope proteins have been well characterised. It is plausible there is significant overlap between nuclear envelope and endoplasmic reticulum proteins due to the connections between these two membrane systems.

#### 3.3.1 Nuclear pore - GO:0005643

Proteins within the nuclear pore complex give a characteristic signal distribution with small puncta covering the entire outside of the nucleus at all stages of the cell cycle. Images captured with the focal plane directly through the centre of the nucleus reveal the nuclear envelope-confined nature of nuclear pores. **Example protein: Nuclear pore protein 152, NUP152** [28] (Fig. 2, Fig. 3, Fig. 4).

#### 3.3.2 Nuclear lamina - GO:0005652

The nuclear lamina is a cytoskeletal structure supporting the nuclear envelope. A nuclear lamina protein would be expected to have a concentration of signal at the nuclear periphery with a punctate/patchy pattern. To date, the only candidate nuclear lamina protein characterised had a localisation similar to nuclear pores, perhaps instead suggesting a role in nuclear lamina-nuclear pore interaction [29].

## 4 Cytoplasm - GO:0005737

This includes the entire plasma membrane bound contents of the cell, excluding the nuclear lumen and the flagellar cytoplasm. This comprises both small organelles and soluble components of the cytoplasm. Cytoplasmic proteins give a whole cell signal that is excluded from the nucleus and the flagellum. A cytoplasmic signal can also have a range of different textures such as smooth, patchy, reticulated and punctate; these correspond to sub-structures within the cytoplasm. **Example protein: S11, a ribosome subunit**, the ortholog of which localises to the cytoplasm in yeast [15]. (Fig. 2, Fig. 3, Fig. 4). Note that the parental cell line, which does not express any fluorescent protein, tends to have a weak reticulated or punctate cytoplasmic signal.

### 4.1 Glycosome - GO:0020015

These are small slightly elongated membrane bound organelles, related to peroxisomes, found throughout the cytoplasm [30,31]. Proteins in glycosomes give a characteristic signal that looks like a short line or elongated point, and glycosomes tend to cluster into groups away from the nucleus and flagellar pocket in *T. brucei*. In *Leishmania* the position of the glycosomes is similar; however, the signal is more rounded. **Example protein: Glycosomal glyceraldehyde 3-phosphate dehydrogenase, GAPDH** [32,33] (Fig. 2, Fig. 3, Fig. 4).

### 4.2 Acidocalcisome - GO:0020022

This is a small spherical membrane bound organelle, which contains very high concentrations of calcium and sodium ions. They are expected to have little luminal protein content, with most acidocalcisome proteins expected to be transporters. An acidocalcisome protein signal is characteristically composed of multiple point-like or circular foci that cluster away from the nucleus and flagellar pocket. **Example protein: Vacuolar iron transporter 1, VIT1** [34] (Fig. 2, Fig. 3). When VIT1 tagged with mNeonGreen at its C-terminus is expressed in *L. mexicana* amastigotes it does not obviously localise to acidocalcisomes; instead it has a reticulated fluorescent signal, which we think is likely to be spurious (Fig. 4). This is potentially due to expression with an endogenous 3′ UTR being particularly critical in this cell type.

### 4.3 Lipid droplet - GO:0005811

This is a storage organelle for lipids and lipid soluble molecules, which has little internal protein content, though has some surface-associated proteins. A lipid droplet protein is characterised by multiple circular signal foci throughout the cytoplasm, which are larger than acidocalcisomes or RNA granules [35]. The size and number of lipid droplets is dependent on the nutritional status of the cell [35]. In *Leishmania* promastigotes for some foci the signal appeared ring-like, which is plausible for larger lipid droplets if the protein is only associated with the droplet periphery. In *Leishmania* amastigotes there was no observable signal for our example protein. **Example protein: Lipid droplet kinase, LDK** [36] (Fig. 2, Fig. 3).

### 4.4 RNA granules

RNA granules are non-membrane bound structures within the cytoplasm, which are sites for the storage, processing and degradation of RNA. RNA granules are highly dynamic and complex, and there are multiple different types of RNA granule, such as P-bodies and stress granules, which appear under different cellular conditions. An RNA granule protein localisation is characterised by multiple point-like foci of variable sizes throughout the cytoplasm and the number, size and distribution depends on their type and the precise state of the cell. We have observed this to have some variability, which we presume results from the level of stress arising from the precise time the live cell was adhered to the slide before imaging. These different granule types can be distinguished by co-localisation with a known marker. **Example protein: SCD6** [37] (Fig. 2, Fig. 3, Fig. 4).

### 4.5 Endocytic

In *T. brucei* and *L. mexicana*, all exocytic/endocytic activity occurs at the flagellar pocket. The position of the single lysosome and single Golgi apparatus means that almost all exocytic/endocytic traffic is concentrated in the region between the flagellar pocket and nucleus. One exception to this is the *Leishmania* lysosome which extends from near the flagellar pocket to beyond the nucleus, and there may therefore be some associated endocytic traffic in this region. The exocytic/endocytic apparatus includes specific compartments such as the early/late/recycling endosomes but we have chosen, for simplicity, to use a higher-level description. Proteins in exocytic/endocytic apparatus give signals as either a single focus or a complex of multiple foci between the flagellar pocket and nucleus. More detail about the exact compartment(s) from which the signal is originating can be determined by co-localisation with known marker proteins. **Example protein: RAB5A** [38] (Fig. 2, Fig. 3, Fig. 4).

#### 4.5.1 Lysosome - GO:0005764

This is a membrane bound organelle that is a terminal destination of the endocytic pathway and is responsible for the breakdown of many different cellular or endocytosic substrates. The *T. brucei* lysosome is found between the flagellar pocket and nucleus towards the ventral side; signal from a lysosome protein therefore appears as a small focus positioned relatively close to the posterior side of the nucleus. In contrast, the lysosome in *L. mexicana* is an elongated tube that runs along the anterior-posterior axis of the cell from a multivesicular complex close to the flagellar pocket, past the nucleus, then terminating in the posterior half of the cell. Signal from a *Leishmania* lysosome protein therefore appears as a line that runs from anterior, near the pocket, beyond the nucleus and towards the posterior. **Example protein: cysteine peptidase A, CPA** [39,40] (Fig. 2, Fig. 3, Fig. 4).

#### 4.5.2 Golgi apparatus - GO:0005794

Signal from a Golgi apparatus or endoplasmic reticulum exit site protein appears as a short line positioned near the flagellar pocket. In *T. brucei* the Golgi apparatus is asymmetrically positioned towards the flagellar (dorsal) side of the cell, near the start of the flagellum attachment zone and the neck of the flagellar pocket, and is oriented parallel to the flagellum. In *Leishmania* the Golgi apparatus lies parallel to the flagellar pocket. It is consistently positioned on one side of the pocket; however, a dorsal-ventral axis in these cells is not readily established from only light microscopy. The Golgi has differences between the cis and trans compartment composition, potentially enabling the localisation of proteins to particular sub-domains. **Example protein: GRASP** [[41], [42], [43]] (Fig. 2, Fig. 3, Fig. 4).

#### 4.5.3 Endoplasmic reticulum (ER) - GO:0005783

An endoplasmic reticulum protein gives a reticulated cytoplasmic signal, often with a perinuclear (i.e. nuclear envelope) signal. This signal appears somewhat similar to that of a protein localised to the mitochondrion; however, its tubules are thinner, it typically has areas of more diffuse signal corresponding to the cisternae and has minimal signal around the kinetoplast. Specialised sub-domains of the ER are known, particularly in *T. brucei*, including flagellum attachment zone [44] and flagellar pocket [45] associated sub-domains. It is likely that some ER proteins are enriched in particular sub-domains (e.g. flagellum attachment zone ER-enriched VAP [46]), but few such examples have yet been analysed. **Example protein: serine palmitoyltransferase, SPT**, the ortholog of which localises to the ER in humans [14] and yeast [15] (Fig. 2, Fig. 3, Fig. 4). A weak or ambiguous ER localisation may be annotated cytoplasm with the modifier reticulated; see the use of modifiers below.

### 4.6 Mitochondrion - GO:0005739

*T. brucei* and *Leishmania* have a single reticulated mitochondrion which extends throughout the cytoplasm, from the posterior to the anterior ends of the cell. The reticulation/tubules of the mitochondrion surround the kinetoplast (which lies within the mitochondrion) and are thicker than those of the endoplasmic reticulum. The double membrane of the mitochondrion means there are two sub-compartments, the inter-membrane space and the matrix, in addition to the two membranes. It may be the case that these give characteristic signals. **Example protein: Translocase of the inner membrane 17, TIM17**, a well-conserved mitochondrion translocase protein (Fig. 2, Fig. 3, Fig. 4). As for the ER, a weak or ambiguous mitochondrion localisation may be annotated cytoplasm with the modifier reticulated; see the use of modifiers below.

#### 4.6.1 Kinetoplast - GO:0020023

The kinetoplast is disc-shaped and lies next to the basal body with its long axis perpendicular to the orientation of the flagellum. A kinetoplast protein localisation can be identified by co-localisation of signal with stained kinetoplast DNA. **Example protein: PIF1-like helicase 8, PIF8** [47] (Fig. 2, Fig. 3, Fig. 4).

#### 4.6.2 Tripartite attachment complex (TAC)

This is the transmembrane cytoskeletal complex that links the kinetoplast to the basal body and crosses the inner and outer mitochondrial membranes. Proteins localised to the TAC give a small focus of signal extremely close to the kinetoplast on the side of the flagellar pocket, basal body and flagellum. The TAC has an anisotropic multi-layered structure with distance from the kinetoplast corresponding to position in this structure [48]. **Example protein: P197** [49] (Fig. 2, Fig. 3, Fig. 4).

#### 4.6.3 Antipodal sites

These sites define the two poles of the kinetoplast disc and are associated with the addition of new minicircles during mitochondrial S phase. Antipodal site proteins give two points of signal at the tips of the kinetoplast along its long axis, oriented perpendicular to the flagellum. Given that these are the site of minicircle addition during mitochondrial S phase, proteins may only localise to this structure at some stages of the cell cycle. **Example protein: Mitochondrial RNA binding protein 38, RBP38** [50] (Fig. 2, Fig. 3, Fig. 4).

## 5 Flagellum and associated structures

### 5.1 Flagellum - GO:0005929

The morphology and position of the flagellum is one of the defining features of the different trypanosomatid morphology classes [7]: *T. brucei* procyclic forms are trypomastigote with the flagellum running laterally attached to the side of the cell towards the anterior end of the cell, where it overhangs the cell by a short distance. In *Leishmania* procyclic promastigotes the flagellum protrudes from the anterior of the cell with a short stretch of lateral attachment within the flagellar pocket. In morphologies with a motile flagellum, signal localisations can normally be assigned with confidence to a flagellum sub-structure. However, *Leishmania* amastigotes have a short, immotile flagellum that barely extends beyond the cell body, has a collapsed 9 + 0 (9v) axoneme and no paraflagellar rod [51,52]. Several flagellum structures are missing and this difference in architecture leads to differences in the localisation of many proteins in the flagellum and associated structures.

#### 5.1.1 Flagellar cytoplasm - GO:0097014

This is the membrane bound contents of the flagellum. A flagellar cytoplasm protein would give signal visible as a flagellar localisation which is more diffuse and/or wider than an axonemal or paraflagellar rod signal. It is plausible that some proteins may be concentrated in the flagellar cytoplasm relative to the rest of the cytoplasm, through the action of the transition zone or the proposed ‘ciliary pore complex’; however to date there are no clear examples of proteins concentrated in the flagellar cytoplasm in trypanosomatids.

#### 5.1.2 Axoneme - GO:0005930

The axoneme is the microtubule based cytoskeleton of the flagellum that extends from the basal body to the distal tip of the flagellum. Axoneme protein signals typically extend from close to the kinetoplast, through the flagellar pocket to the distal end of the flagellum. Some structures including the central pair, inner dynein arms and all structures on the distal microtubule doublets are absent in *Leishmania* amastigotes [51]. **Example protein: Outer arm dynein β, OADβ** [53] (Fig. 2, Fig. 3).

##### 5.1.2.1 Basal body - GO:0036064

The basal body nucleates the axoneme in close proximity to the kinetoplast. A basal body protein localisation is identifiable by a single point of signal at the base of the flagellum extremely close to the kinetoplast and next to the flagellar pocket. Trypanosomatids have a mature basal body subtending an axoneme and adjacent to this an immature pro-basal body which will nucleate the new flagellum in the next cell cycle. As such many basal body proteins are also found in the pro-basal body. **Example protein: SAS6** [49,54] (Figs. 2–4).

##### 5.1.2.2 Pro-basal body

This is an immature basal body that has not yet nucleated a flagellum and is found tethered to an adjacent basal body. Signal from a pro-basal body protein would be difficult to distinguish from a basal body signal without evidence from co-localisation, so in practice a pro-basal body protein localisation is only identifiable if the protein is also present in the basal body. In this case this gives two points of signal between the kinetoplast and the flagellar pocket, separated perpendicular to the orientation of the flagellum. It is likely that there are also proteins that link the basal and pro-basal body, which would give a single point signal between these two structures [55]. **Example protein: SAS6** [54] (Fig. 2, Fig. 3, Fig. 4).

##### 5.1.2.3 Transition zone - GO:0035869

This is a small, specialised region of the flagellum bounded at the proximal end by the basal body and at the distal end by the start of the axoneme proper (the start of axonemal central pair microtubules) [56,57] A transition zone protein gives a single dot of signal similar to that of a basal body protein; however, the signal often appears in the portion of the flagellum within the flagellar pocket, more distal than the basal body. **Example protein: Transition zone protein 50, TZP50** [58] (Fig. 2, Fig. 3, Fig. 4).

##### 5.1.2.4 Flagellar tip - GO:0097542

This is the distal tip of the axoneme; proteins localised here give a characteristic point of signal at the tip of the flagellum. This is distinct from signal from proteins localised at the distal end of the flagellar membrane, which typically have a horseshoe shaped signal around the tip of the flagellum. **Example protein: Axoneme capping structure 2, ACS2** [59] (Fig. 2, Fig. 3, Fig. 4).

#### 5.1.3 Flagella connector – GO:0120118

This structure is only present in *T. brucei* and connects the tip of the growing new flagellum to the side of the old flagellum. Proteins localised to the flagella connector give a dot at the tip of the new flagellum with the signal progressing along the side of the old flagellum as the cell progresses through the cell cycle. For some flagella connector proteins a signal may also been seen on cells that have just completed cytokinesis either at the flagellum tip or mid-way up the flagellum depending on whether the cells inherited the new or the old flagellum. **Example protein: Flagella connector protein 1, FCP1** [59,60] (Fig. 5).

#### 5.1.4 Paraflagellar rod (PFR) – GO:0097740

This is an extra-axonemal structure of comparable size to the axoneme itself, and runs parallel to the axoneme for most of the length of the flagellum. A paraflagellar rod protein gives an axoneme-like signal; however, at its proximal end the signal does not extend into the flagellar pocket and the signal fades towards the distal end of the flagellum. The paraflagellar rod is not present in *Leishmania* amastigotes [61]. **Example protein: Paraflagellar rod 2, PFR2** [62,63] (Fig. 2, Fig. 3).

#### 5.1.5 Intraflagellar transport (IFT) particle - GO:0030990

In long exposure images, signal from intraflagellar transport proteins gives patchy flagellum localisation seen with a strong signal in the basal body region. The patchy signal within the flagellum arises from the movement of the protein as the image is acquired. In short exposure images (200 ms or less), the IFT particles appear as point-like foci or short lines parallel to the flagellum and in videomicrographs motion of individual intraflagellar transport particles can be observed. In *L. mexicana* amastigotes a signal is observed at the base of the flagellum but no patchy signal is seen within the flagellum. **Example protein: Intraflagellar transport 172, IFT172** [[64], [65], [66]] (Fig. 2, Fig. 3, Fig. 4).

### 5.2 Flagellar membrane - GO:0060170

This is the specialised domain of the cell membrane that encloses the flagellum. Signal from a flagellar membrane protein appears like two closely spaced parallel lines along the outside edges of the flagellum, arising from the ‘edge effect’ of the membrane localisation. The signal appears to penetrate a short distance into the cell body, to the base of the flagellar pocket near the kinetoplast. **Example protein: Flabarin-like, FlabarinL (*T. brucei*)** [67] **and Flabarin (***L.* ***mexicana*)** [68] (Fig. 2, Fig. 3). When Flabarin is expressed in *L. mexicana* amastigotes tagged with mNeonGreen at its C-terminus it does not localise to the flagellum but instead has a reticulated fluorescent signal – a result which carries the above rehearsed caveats (Fig. 4).

### 5.3 Flagellar pocket - GO:0020016

This term refers to the entire flagellar pocket, the invagination of the cell membrane at the base of the flagellum. In *T. brucei* it is identifiable in phase contrast images as a bright spot near the kinetoplast, but tends not to be easily visible in phase contrast images of *Leishmania.* The flagellar pocket is the sole site for exocytosis/endocytosis and also has a complex set of associated cytoskeletal structures. Normally, it is possible to identify with which sub-structure of the flagellar pocket a protein is likely associated.

#### 5.3.1 Flagellar pocket membrane - GO:0020018

The flagellar pocket membrane is the specialised domain of the cell membrane that encompasses the flagellar pocket; together with the flagellar membrane and pellicular membrane it makes up the entire cell membrane. It is visible as a smooth ring of signal near the kinetoplast. In *T. brucei* this signal lies around the phase bright flagellar pocket. Currently, there are no proteins with a published convincing flagellar pocket or flagellar pocket membrane (see below) localisation for procyclic form *T. brucei* or *Leishmania*. Some proteins are known in the *T. brucei* bloodstream form [69].

#### 5.3.2 Flagellar pocket neck complex

We suggest this term for the complex of interlinked cytoskeletal structures around the flagellar pocket neck and including the flagellar pocket collar and hook complex. It is intimately linked with the microtubule quartet and flagellum attachment zone (see below). We anticipate the collar and hook complex to be the only components of the flagellar pocket neck complex; however, electron microscopy analysis shows that this region is complex and *T. brucei* and *Leishmania* have several differences. There may be as yet uncharacterised divergent specialised structures [39,41,65]. Proteins within this complex region give a structured signal around the flagellar pocket and/or the exit of the flagellum from the pocket.

##### 5.3.2.1 Flagellar pocket collar - GO:1990900

This ring or horseshoe-shaped structure defines the boundary between the flagellar pocket and the pocket neck. Signal from proteins localised to the pocket collar appears as a short line perpendicular to the flagellum at the distal side of the flagellar pocket, but can also appear as a ring (particularly in *T. brucei*) depending on cell orientation. The only *bona fide* collar protein known is BILBO1 [70]; however, expression of BILBO1 with a fluorescent protein tag over an extended period (>48 h) causes growth arrest in *T. brucei* [70] and, to date, it has not been possible to determine the localisation of this protein using a fluorescent protein rather than an epitope tag in *Leishmania* [42].

##### 5.3.2.2 Hook complex – GO:0120120

The hook complex (previously termed the bilobe) describes a region of cytoskeletal structures at the distal side of the flagellar pocket as the flagellum exits the cell body. Hook complex proteins can give a hook, short line or bilobed shaped signal near the flagellar pocket neck. In *T. brucei* this is the start of the extended flagellum attachment zone. **Example protein: LRRP1** [71] (Fig. 2). The hook complex has not been characterised in *Leishmania.* The *Leishmania* LRRP1 homolog localised to the flagellar pocket region but an in-depth analysis of its localisation was beyond the scope of this study (Fig. 3, Fig. 4).

#### 5.3.3 Microtubule quartet

These four specialised microtubules nucleate near the basal body and then run around the flagellar pocket passing through a gap in the flagellar pocket collar at the distal end of the flagellar pocket. In *T. brucei* they continue on to the anterior end of the cell parallel to the extended flagellum attachment zone [44,72]. In *L. mexicana* there are additional microtubules nucleating near the flagellar pocket [42,73]. To date, only proteins that localise to the proximal region of the microtubule quartet as it loops around the flagellar pocket have been identified. Signal from proteins localised to this section of the microtubule quartet curves around the flagellar pocket. **Example protein: SPEF1** [49] (Fig. 2). In *L. mexicana* SPEF1 also appears to localise to the cytoplasmic microtubule in addition to the microtubule quartet (Fig. 3, Fig. 4).

#### 5.3.4 Flagellum attachment zone (FAZ) – GO:0120119

This structure connects the flagellum to the cell body, traversing both the flagellum and pellicular membranes. Unlike most structures, the FAZ exhibits major differences between *T. brucei* and *L. mexicana*. In trypomastigotes, including *T. brucei*, the FAZ extends along the entire length of the flagellum that is laterally attached to the cell body. Proteins localised to the FAZ give a linear signal that begins as the flagellum exits the cell body and runs to the anterior end of the cell body. This signal is positioned between the flagellum and the cell body so appears offset in comparison to an axoneme or paraflagellar rod signal. In *Leishmania* the FAZ is restricted to the flagellar pocket neck with some specific elaborations. *Leishmania* FAZ proteins can give signal shaped like a short line, a short line with a ring or a ring/horseshoe around the flagellum exit point [42]. The *Leishmania* FAZ is likely more similar to the ancestral trypanosomatid, with the extended FAZ of *T. brucei* an innovation in the *Trypanosoma* lineage [74]. **Example protein: Flagellum attachment zone 1, FAZ1** [44] (Fig. 2, Fig. 3, Fig. 4).

#### 5.3.5 Cytostome - GO:0031910

Neither *Leishmania* nor *T. brucei* have a cytostome, but as many trypanosomatids do we have included this structure here for completeness. *T. cruzi* has a cytostome [75] and it is likely the ancestral trypanosomatid also had a cytostome [74].

## 6 Cell cortex - GO:0005938

The cell cortex includes the entire cell surface and associated structures.

### 6.1 Plasma membrane - GO:0005886

The plasma membrane is the entire cell membrane including the pellicular, flagellar and flagellar pocket membrane. Signal from a plasma membrane protein outlines the cell body, flagellum and flagellar pocket, with a clear ‘edge effect’.

#### 6.1.1 Pellicular membrane

This sub-domain of the plasma membrane excludes the flagellar and flagellar pocket membranes. The pellicular membrane protein signal outlines the entire cell body with a clear ‘edge effect’, with no signal on the flagellar pocket or flagellum. Membrane protein signal tends to be uniform over the entire cell surface. **Example protein: Glucose transporter 2 A, THT2 A** [5] (Fig. 2, Fig. 3). THT2 A is not expressed in *L. mexicana* amastigotes [76].

#### 6.1.2 Cortical cytoskeleton - GO:0030863

Proteins localised to the cortical cytoskeleton give a signal similar to the pellicular membrane; outlining the cell body with a clear ‘edge effect’. However, unlike the pellicular membrane localisations, the signal is typically non-uniform. It is often excluded from the posterior tip of the cell, and can sometimes be excluded from other areas. **Example protein: Whole cell body, WCB** [77] (Fig. 2, Fig. 3, Fig. 4).

#### 6.1.3 Cell tip - GO:0051286

This is the extreme end of the cell body, either the anterior or posterior end of the cell. Cell tip proteins may give signal seen as a dot or a region at and/or near the anterior or posterior of the cell body. Signal is typically seen only at either the posterior or anterior. **Example protein: XMAP215** [19] (Fig. 2, Fig. 3, Fig. 4).

#### 6.1.4 Cleavage furrow – GO:0032154

This is the furrow formed as the cell undergoes cytokinesis, progressing from the cell anterior to posterior. It is only present in cells undergoing cytokinesis. Cleavage furrow proteins give signal along the line of cytokinesis, typically at the leading edge of the advancing furrow.

#### 6.1.5 Midbody – GO:0030496

This is the structure that transiently links the daughter cells at the final stages of cytokinesis [19] and is present only at the very latest stages of cytokinesis. Midbody proteins would give signal visible as a thin line connecting the posterior ends of the two daughter cells at the end of cytokinesis, but no proteins which localise only to this structure have yet been identified.

## 7 Localisation ontology use in the TrypTag project

The *T. brucei* localisations shown here form the basis of the annotation system for the TrypTag project [13] and provide a reference for determining protein localisation to an organelle with confidence from fluorescent signal. Terms are used in a strictly additive manner, meaning many proteins will have multiple annotation terms. For example, “cytoplasm, flagellar cytoplasm, nuclear lumen” describes a protein that localises throughout the cell as a soluble protein. “flagellum tip, basal body, pro-basal body” describes a protein which localises to those three distinct structures.

These localisation annotations fit into a hierarchical system with complex organelles and structures made up of a set of sub-annotations. For example, the nucleus has up to four levels of hierarchy with the overarching term being nucleus, which is then divided into nuclear lumen and nuclear envelope. Within the nuclear lumen there is the nucleoplasm, nucleolus, spindle and finally within the spindle there are the spindle poles and the kinetochores. An annotation of “kinetochore” therefore implies this protein also localises to the spindle, within the nuclear lumen of the nucleus.

This system also allows for ambiguity when a fluorescent signal is weak and/or unconvincing. For example, “nucleus” indicates the protein may localise within any of the sub-annotations and could be used for a weak but clearly nuclear signal. Similarly, “flagellum” could be used as an annotation with confidence for a weak or ambiguous axoneme or flagellar membrane fluorescent signal.

For TrypTag, we are using a system of modifiers that identify qualitative properties of the signal: Relative strength of the signal (strong, weak) for proteins which localise to multiple organelles, whether a signal appears in a subset of cells or at particular cell cycle stages (<10%, 25%, 50%, 75% or cell cycle dependent – only observed in cells at a specific point in the cell cycle), its position along one of the reference axes (anterior, posterior, proximal, distal, end) and any texture/structure in the signal (reticulated, point, points, patchy, peripheral, region) [13]. For example, “cell tip [posterior]” indicates a protein localised to the posterior pole of the cell. “nucleolus [peripheral, patchy]” indicates a protein localised to the nucleolus, but restricted to patches around its edge. These modifiers are subjective, but provide a useful qualitative descriptor for complex localisations.

Some regions of the cell are complex and there are multiple possible localisations. In this case all plausible annotations are given: “cell tip [anterior], flagellum attachment zone [distal, end]” indicates a protein localisation in the anterior cell tip and/or the extreme distal end of the flagellum attachment zone. It may be the case that some of these annotations are effectively synonyms.

The system of modifiers also allows for some degree of explanation in cases where fluorescent signal was insufficiently convincing to assign a highly specific annotation. In these situations, an annotation one step up the hierarchical system with an explanatory modifier can be used. For example, “cytoplasm [reticulated]” would be used if the fluorescent signal is clearly reticulated throughout the cytoplasm, but with insufficient clarity as to whether it arose due to an endoplasmic reticulum or mitochondrial protein localisation.

## 8 Protein identifiers of cellular landmarks

Having defined a coherent set of GO terms and a hierarchy we sought to provide a cohort of the best defined proteins that will act as a collection of identifiers for particular organelles and structures within kinetoplastid parasites (Table 1). This resource can be used in many ways – for individual or collective marking of cells within a variety of experiments. We have only included proteins whose localisation provides a robust and reproducible definition of the organelle or structure. Making individually or multiply tagged cell lines is a rather trivial exercise, but one that is likely to facilitate the interpretation of many experiments in these systems.

**Table 1:** XXX.

	Localisation ontology term	Gene ontology ID	Protein name	Terminus	Gene ID		Notes
					*T. brucei*	*L. mexicana*	
Nucleus	nucleoplasm	GO:0005654	H3	N	Tb927.1.2430	LmxM.10.0990	
	nucleolus	GO:0005730	DEAD/H	N	Tb927.5.1560	LmxM.15.0130	
	spindle	GO:0005819	KIN5	C	Tb927.3.2020	LmxM.25.1950	
	kinetochore	GO:0000776	KKT1	N	Tb927.10.6330	LmxM.36.1900	
	nuclear pore	GO:0005643	NUP152	N	Tb927.10.9650	LmxM.36.4270	
Cytoplasm	cytoplasm	GO:0005737	S11	N	Tb927.1.3180	LmxM.20.1650	
	glycosome	GO:0020015	GAPDH	N	Tb927.6.4300	LmxM.29.2980	
	acidocalcisome	GO:0020022	VIT1	C	Tb927.3.800	LmxM.27.0210	Not suitable for amastigotes
	lipid droplet	GO:0005811	LDK	N	Tb927.11.8940	LmxM.28.2000	Not suitable for amastigotes
	RNA granule	GO:0035770	SCD6	N	Tb927.11.550	LmxM.25.0540	
	endocytic		RAB5A	N	Tb927.10.12960	LmxM.18.1130	
	lysosome	GO:0005764	CPA	C	Tb927.6.1000	LmxM.18.1130	
	Golgi apparatus	GO:0005794	GRASP	C	Tb927.11.2660	LmxM.32.2380	
	endoplasmic reticulum	GO:0005783	SPT	C	Tb927.4.1020	LmxM.33.3740	
Mitochondrion/Kinetoplast	mitochondrion	GO:0005739	TIM17	N	Tb927.11.13290	LmxM.09.1130	
	kinetoplast	GO:0020023	PIF8	C	Tb927.7.1000	LmxM.26.0930	
	tripartite attachment complex	GO:0120121	P197	N	Tb927.10.15750	LmxM.19.1150	
	antipodal sites		RBP38	C	Tb927.8.2740	LmxM.23.0760	
Flagellum	axoneme	GO:0005930	OADβ	N	Tb927.11.3250	LmxM.13.1650	Not suitable for amastigotes
	basal body	GO:0036064	SAS6	N	Tb927.9.10550	LmxM.34.4280	
	transition zone	GO:0035869	TZP50	N	Tb927.10.11840	LmxM.32.1230	
	flagellar tip	GO:0097542	ACS2	C	Tb927.11.450	LmxM.25.0420	
	flagella connector	GO:0120118	FCP1	C	Tb927.8.940		Not present in Leishmania
	paraflagellar rod	GO:0097740	PFR2	N	Tb927.8.4970	LmxM.16.1430	Not present in amastigotes
	intraflagellar transport particle	GO:0030990	IFT172	N	Tb927.10.1170	LmxM.21.0980	
	flagellar membrane	GO:0060170	Flabarin	C	Tb927.11.2400	LmxM.27.1730	Not suitable for amastigotes
Other	hook complex	GO:0120120	LRRP1	C	Tb927.11.8950	LmxM.28.1990	
	microtubule quartet		SPEF1	C	Tb927.4.3130	LmxM.33.1120	
	flagellum attachment zone	GO:0120119	FAZ1	N	Tb927.4.3740	LmxM.33.0690	
	pellicular membrane		THT2A	N	Tb927.10.8510	LmxM.36.6280	Not suitable for amastigotes
	cortical cytoskeleton	GO:0030863	WCB	N	Tb927.7.3550	LmxM.14.1440	
	cell tip	GO:0051286	XMAP215	N	Tb927.6.3090	LmxM.29.1760	

## 9 Conclusions

In the post-genomic era there has been an explosion in the number and size of datasets, which has required a step change in the way we approach their analysis. To aid the analysis of these large datasets we and others have developed technologies that enable the generation of many cell lines expressing fluorescently tagged proteins very rapidly (<2 weeks) [5,78]. To encourage consistency and comparability in the description of these cell lines between experiments and laboratories we have developed the terminology described here. This terminology is carefully designed to be unambiguous, human-readable and searchable. If this terminology became a standard in the field it would be useful for the analysis of many experiments and would be especially appropriate for any protein localisation description - for example, for user-submitted comments concerning protein localisations on the genome database TriTrypDB [79], or for a summary of protein localisations in a paper.

This comprehensive inventory of the organelles and structures of *T. brucei* and *Leishmania* as viewed by fluorescence light microscopy in living cells will hopefully be a useful resource both for the immediate kinetoplastid research field, as it provides a foundation for the analysis of changes in structure in mutants and adaptations in structure in different life cycle stages, and also for other scientists less familiar with these parasites.

## 10 Methods

### 10.1 *T. brucei* and *L. mexicana* cell culture

*T. brucei* procyclic form SmOxP9 [80] cells (derived from TREU 927, expressing T7 RNA polymerase and tetracycline repressor) were grown in SDM-79 media (Life Technologies) with 10% (v/v) FCS (Life Technologies).

Cas9T7 [78] *L. mexicana* (derived from World Health Organisation strain MNYC/BZ/62/M379, expressing Cas9 and T7 RNA polymerase) promastigotes were grown in M199 medium with Earle’s salts and L-glutamine (Life Technologies) supplemented with 10% (v/v) heat inactivated FCS (Life Technologies), 5 mM HEPES·NaOH (pH 7.4), 26 mM NaHCO_3_ and 5 μg/ml haemin at 28 °C. Axenic amastigotes were generated by subculture into Schneider’s Drosophila medium (Life Technologies) supplemented with 20% heat-inactivated FCS and 25 mM MES·HCL (pH 5.5) at 34 °C with 5% CO_2_ for 10 h (for dividing cells) or 72 h (unless otherwise indicated) without subculture.

*T. brucei* and *L. mexicana* cultures were maintained by subculture to achieve a culture density between 1 × 10^6^ and 1 × 10^7^ cells/ml (*T. brucei*) or 1 × 10^5^ and 1 × 10^7^ cells/ml (*L. mexicana*), which gives continuous exponential population growth. Culture density was measured using a CASY model TT cell counter (Roche Diagnostics) with a 60 μm capillary and exclusion of particles with a pseudo diameter below 2.0 μm.

### 10.2 Tagging construct generation and transfection

Constructs for endogenous mNeonGreen tagging for *T. brucei* were generated by long-primer PCR and high-throughput 96-well plate transfection of cells was performed as previously described [81]. The pPOTv7 (mNeonGreen/blast) plasmid was used as the PCR template for generating tagging amplicons. Successful transfectants were selected with 5 μg/ml blasticidin S hydrochloride (Melford Laboratories) 6 h post-transfection. Primers were designed as previously described [5]. These cell lines were generated as part of the TrypTag project [13].

Generation of the *L. mexicana* tagging constructs and sgRNA templates for endogenous mNG tagging were generated by the PCR method as previously described [78] using the pLPOT (mNG/Blast) plasmid as the template. pLPOT is adapted from pPOT and pPLOT with *T. brucei* and *Crithidia fasciculata* 5′ or 3′ untranslated regions (UTRs) and intergenic sequences replaced with complete *L. mexicana* intergenic sequences [53]. Transfection of cells was performed as previously described [5] using the Amaxa Nucleofector-2b. Primers for constructs and sgRNA were designed using LeishGEdit (http://www.leishGEdit.net). Successful transfectants were selected with 5 μg/ml Blasticidin S hydrochloride (Melford Laboratories) 6 to 8 h following transfection.

### 10.3 Fluorescence microscopy

All *T. brucei* and *L. mexicana* cell lines expressing mNeonGreen tagged proteins were examined live. Briefly, parasites were harvested from a log-phase culture by centrifugation at 800 g for 5 min, washed three times in PBS (*L. mexicana*) or vPBS (*T. brucei*, PBS supplemented with 10 mM glucose and 46 mM sucrose) with Hoescht 33342 (1 μg/ml) in the first wash. This washing is necessary to improve adhesion to the glass slide and increase cell density. The cells were re-suspended in 30 μl PBS and 1 to 10 μl was then placed on a microscope slide, a coverslip was applied and immediately imaged using a DM5500 B microscope (Leica Microsystems) with an Andor Neo sCMOS camera and a 63 × NA 1.40 Plan-Apochromat oil immersion objective lens (*T. brucei*) or a Axioimager.Z2 microscope (Zeiss) with a Hamamatsu ORCA-Flash4.0 camera and a 63 × NA 1.40 Plan-Apochromat oil immersion objective lens (*L. mexicana*). *T. brucei* images were captured as part of the TrypTag project and make part of that database.
