# Supplementary material for: Cellular landmarks of Trypanosoma brucei and Leishmania mexicana
Source: Mol Biochem Parasitol. 2019 Jun;230:24–36. doi: 10.1016/j.molbiopara.2018.12.003 (PMC6529878; doi:10.1016/j.molbiopara.2018.12.003)
Supplement: Supplementary file 1 [file mmc1.docx]

Supplementary materials

**pLPOT_plasmid_maps.zip**

GenBank format plasmid maps for the pLPOT series of *Leishmania* long primer PCR-based tagging plasmids. All combinations of eYFP, mNeonGreen and mCherry fluorescent markers with blasticidin, G418 or puromycin selection markers have been generated.

**localisation_ontology_JSON.zip**

Localisation ontology and localisation modifier ontology in JSON (a computer-readable) format.
